# Supplementary figures and images for: A practical application of generative adversarial networks for RNA-seq analysis to predict the molecular progress of Alzheimer's disease
Source: PLoS Comput Biol. 2020 Jul 24;16(7):e1008099. doi: 10.1371/journal.pcbi.1008099 (PMC7406107; doi:10.1371/journal.pcbi.1008099)

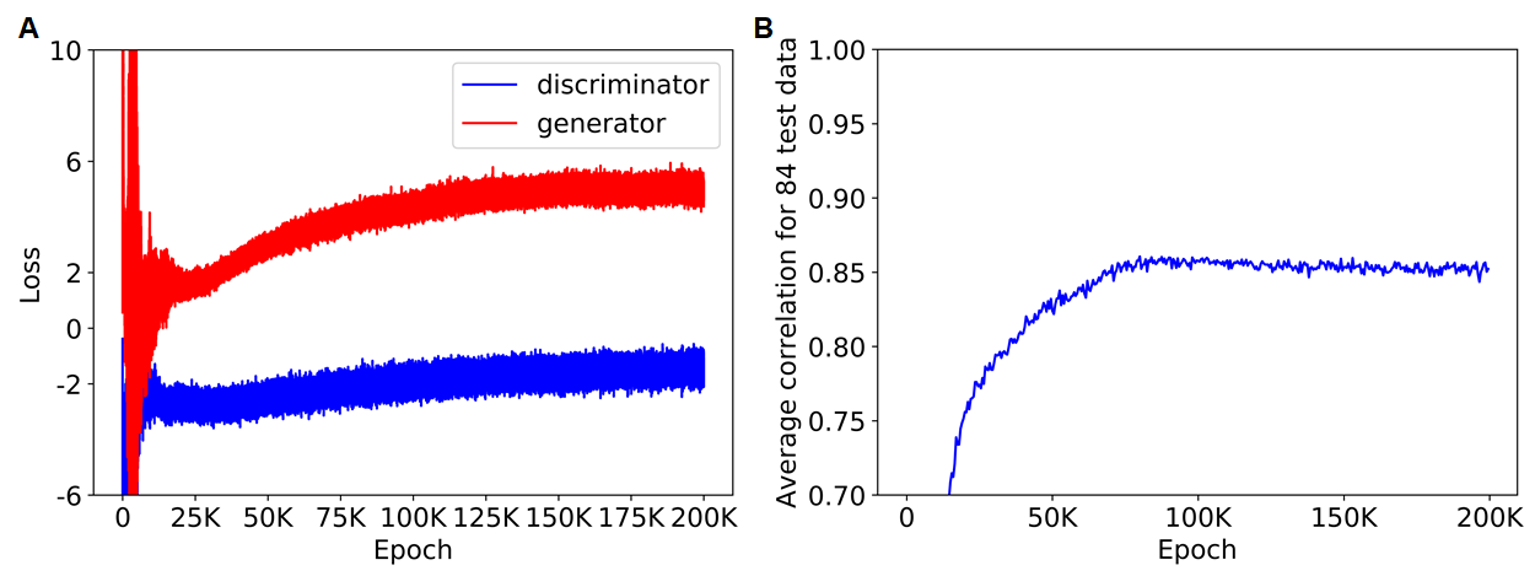

Supplement: S1 Fig — (A) The generator loss (red) and the discriminator loss (blue) throughout the 200k epochs. Training convergence begins after roughly 25k epochs. (B) The average pairwise Pearson correlation between 84 generated data and 84 test data matched by correlation values. The average maximum correlation reached approximately 80k. (TIF) [file pcbi.1008099.s001.tif]

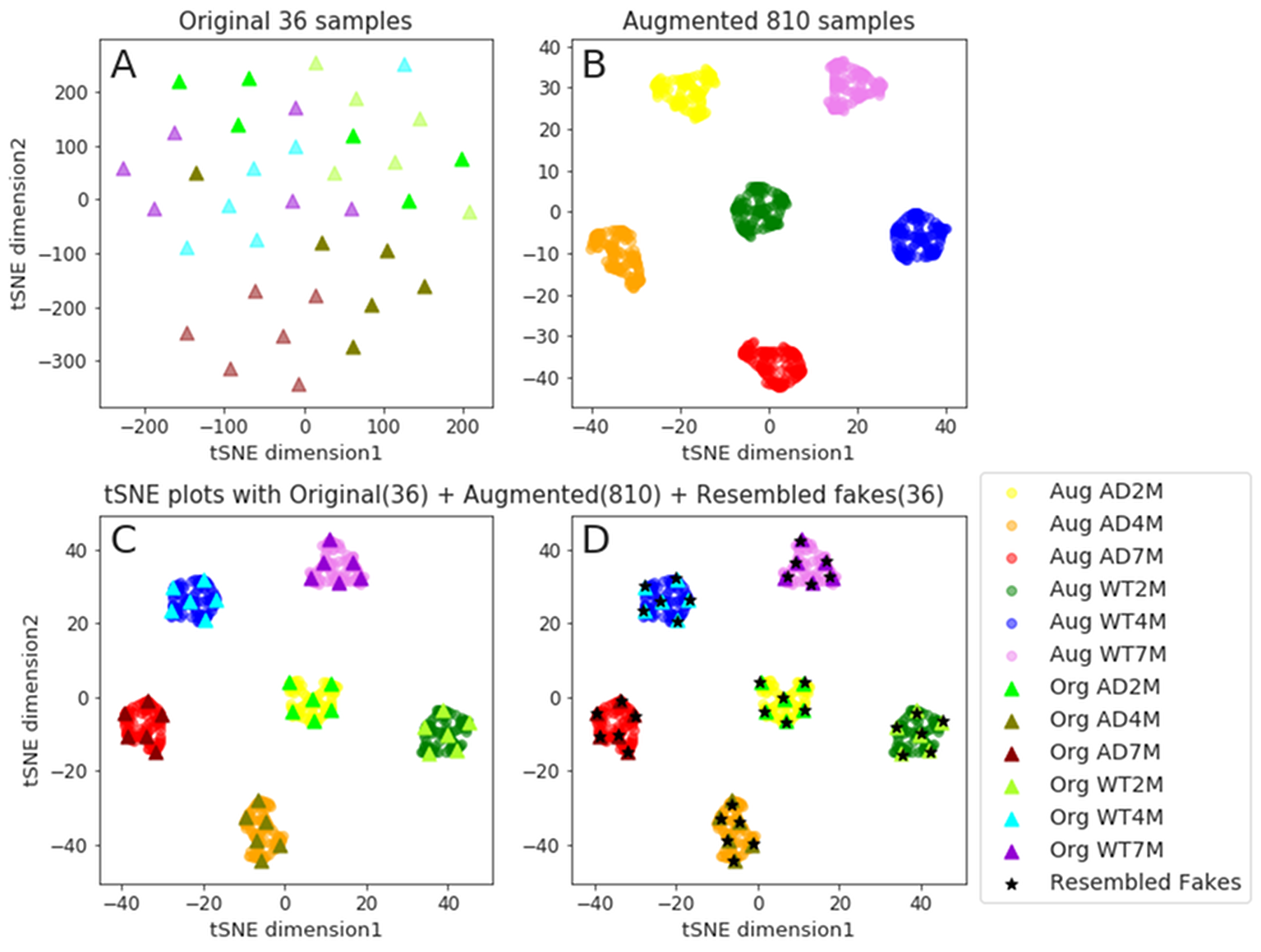

Supplement: S2 Fig — (A) The tSNE plot made by the only original 36 samples shows the scattered sample points without clustering. (B) The tSNE plot made by the only augmented 810 samples shows the clear six groups representing different phenotypes. (C) The tSNE plots of the combined data (the original(36), the augmented(810) and the resembled fakes(36)). The original and the augmented 846 samples are visualized showing excellent clustering of the same groups. (D) The tSNE plot of the combined data including the resembled fakes (black stars) shows well overlaps between the original samples and resembled fakes. (TIF) [file pcbi.1008099.s002.tif]

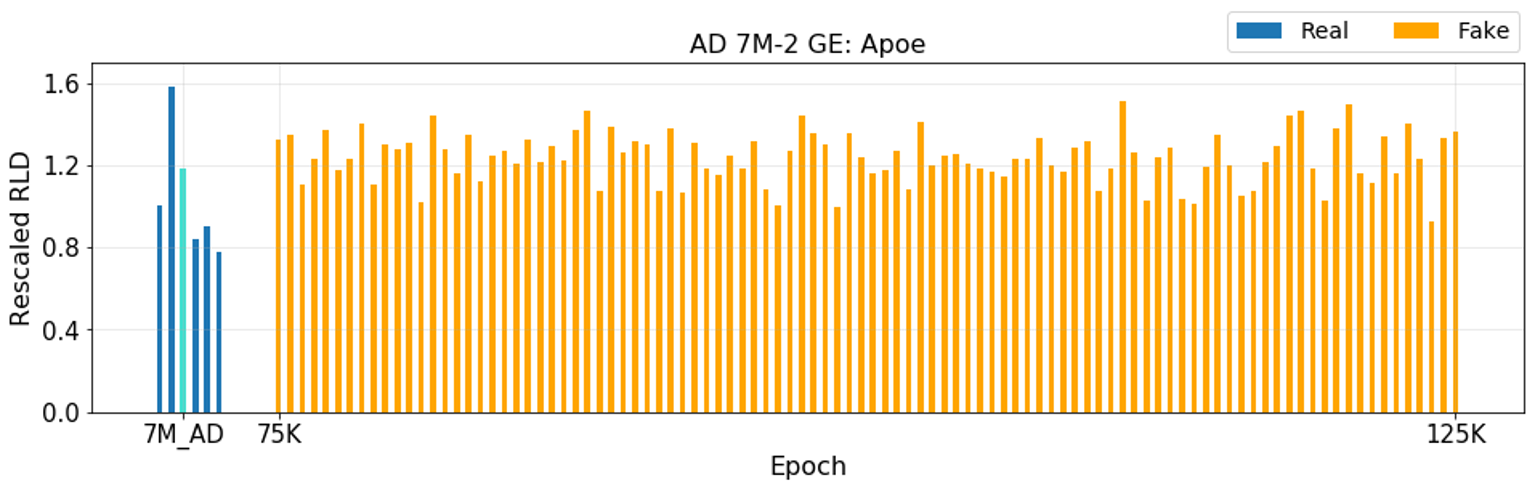

Supplement: S3 Fig — The rescaled RLD of the Apoe gene for the six 7M AD samples (blue and turquoise bars) and the resembled fakes corresponding to the third real data (turquoise bar) during the 75k to 125k epochs. The rescaled RLD values of the Apoe gene show continual variations over the epochs. The standard deviation of the temporal variations of the resemble fakes value is approximately 0.1, which is slightly larger than expected, although the correlation values can range as high as 0.95 ~ 0.99. At first glance, the deviations from the real values appear to be a random process of time. We checked the deviations based on the gene correlation heatmaps (S4 Fig). (TIF) [file pcbi.1008099.s003.tif]

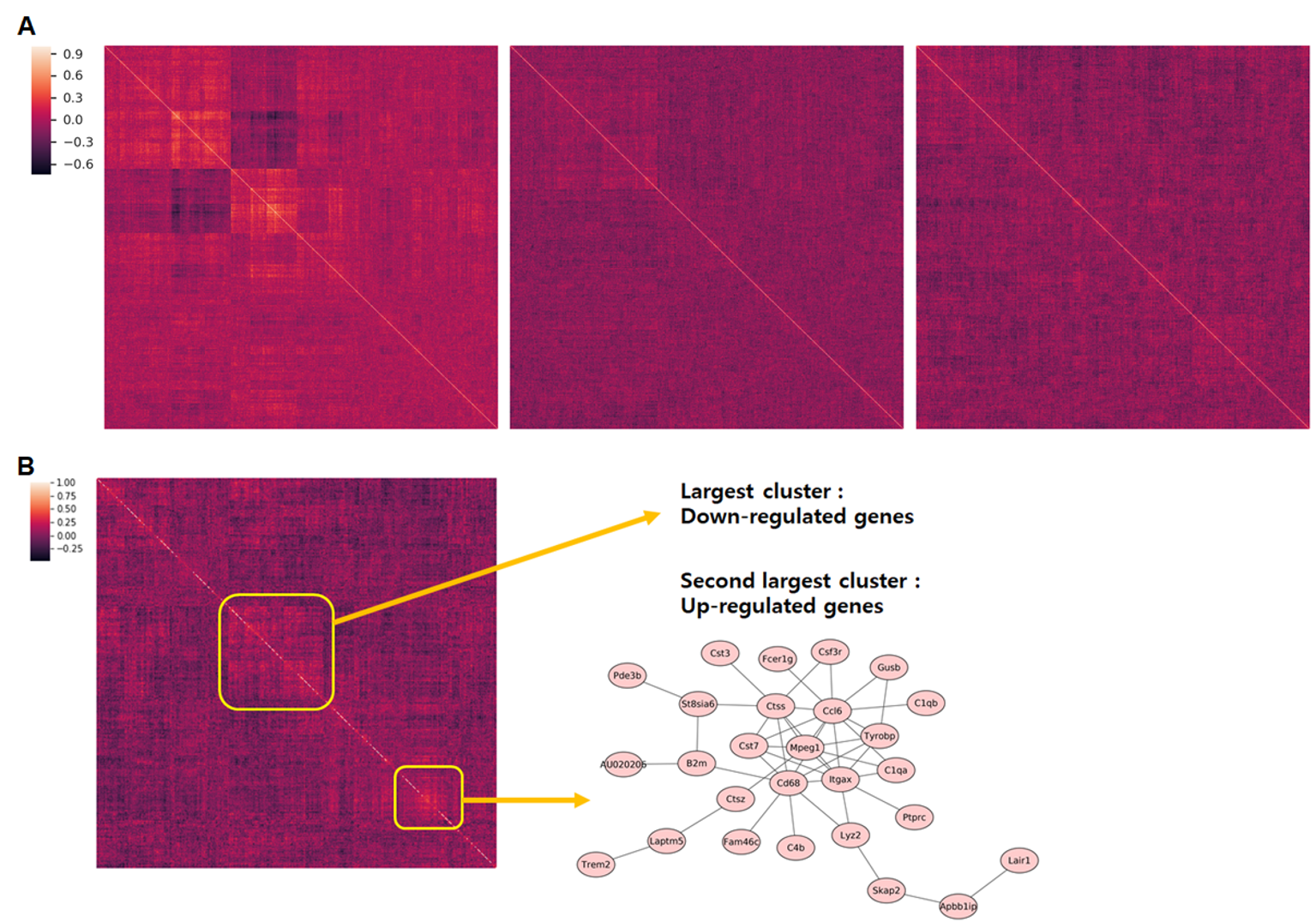

Supplement: S4 Fig — (Top) The correlation heatmaps used to check the collective variations between genes were measured based on the temporal deviations of the 1,208 genes. The images show the correlation heatmaps of the genes obtained by temporal deviations over 7.5k to 125k epochs for a sample in 7M AD (left), the averaged temporal deviations of 6 samples in the 7M AD group (middle) and all 36 samples (right). The heatmap (left) for a sample shows more obvious collective features than do the averaged deviations over a group (middle) or all (right). In this way, we observed genes varying in the same pattern over the epochs, and they represent the properties of a sample rather than the properties between the genes. This indicates that the collective behavior of gene expressions were supposed to be learned and optimized to recognize sample-specific features rather than gene-specific features. (Bottom) The weight parameters of the last layer in the generator are supposed to contain gene-specific features as suggested by Ghahramani et al. in the scRNA-seq study. They suggested this GAN-derived gene association network is corresponding to non-linearly combined co-regulated genes and is distinct from linear and directly correlated regulations of a gene expression profile. Therefore, the correlation heatmap constructed by the weight parameters is predicted to show biological gene associations. The gene-specific heatmap for the 1,208 genes (Fig 2E) appears to be less collective than does the sample-specific heatmap (Top left). However, the collective gene associations in the sample-specific heatmap seems to be too specifically adapted for each sample and do not display appreciably as general gene association networks, while the gene-specific heatmap provides general features that are consistent with known coexpression networks. By using a cutoff of 0.4 in the heatmap, we obtained gene network clusters (left) and present the second-largest cluster to show the core upregulated genes related to t [file pcbi.1008099.s004.tif]

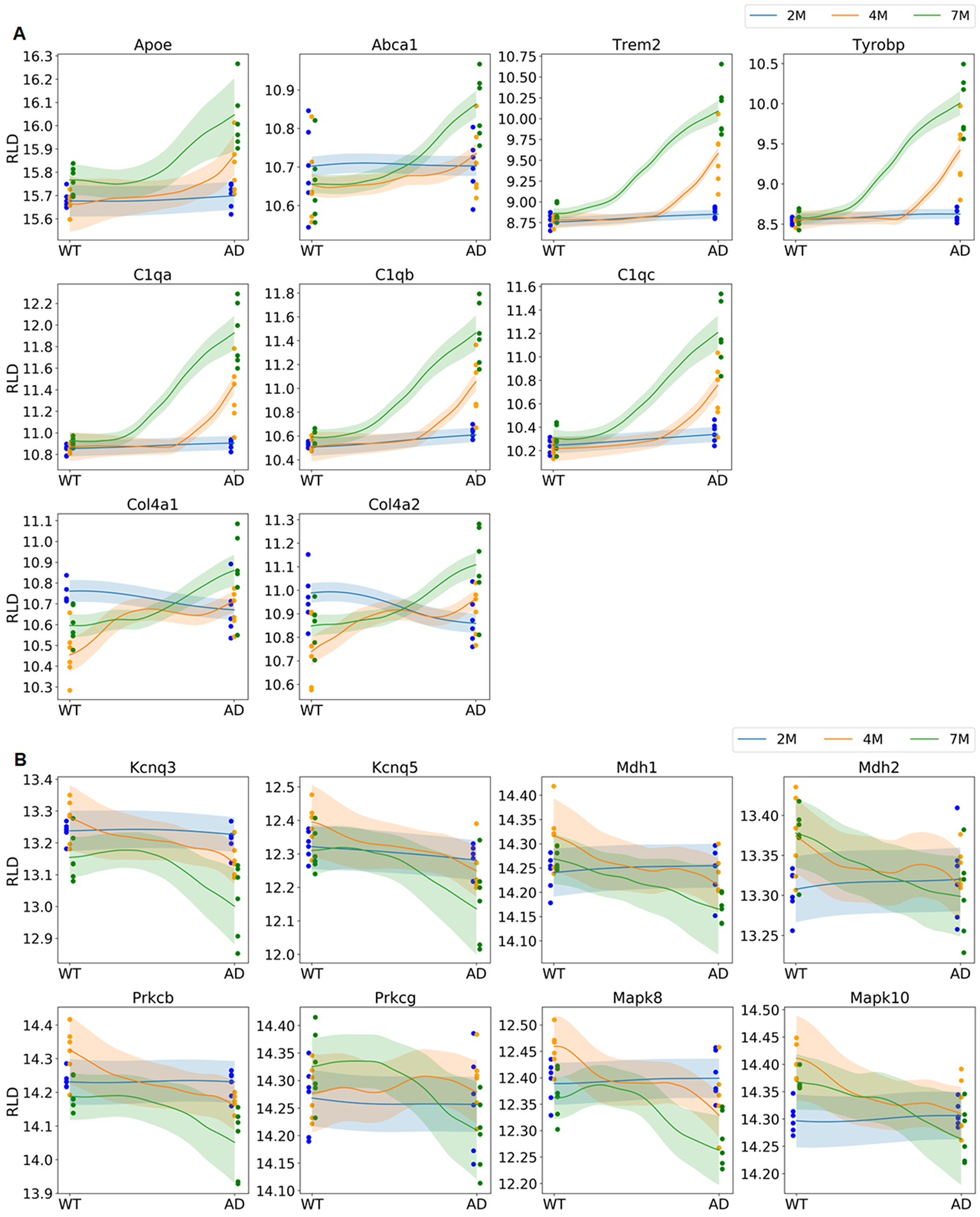

Supplement: S5 Fig — The vertical curve widths represent the standard deviations of the latent interpolations over the 75k to 125k epochs. All the starting and ending points of the curves are in the middle of the original data points, indicating that the latent space interpolation works quite well. The plots show little change in 2M, which indicates that there is little difference between the AD and WT. However, we can observe differences in 4M and 7M. Some genes, such as Apoe and Abca1, have been shown to exhibit a delayed increasing pattern with little change during the first half and a sharp increase during the last half. (TIF) [file pcbi.1008099.s005.tif]

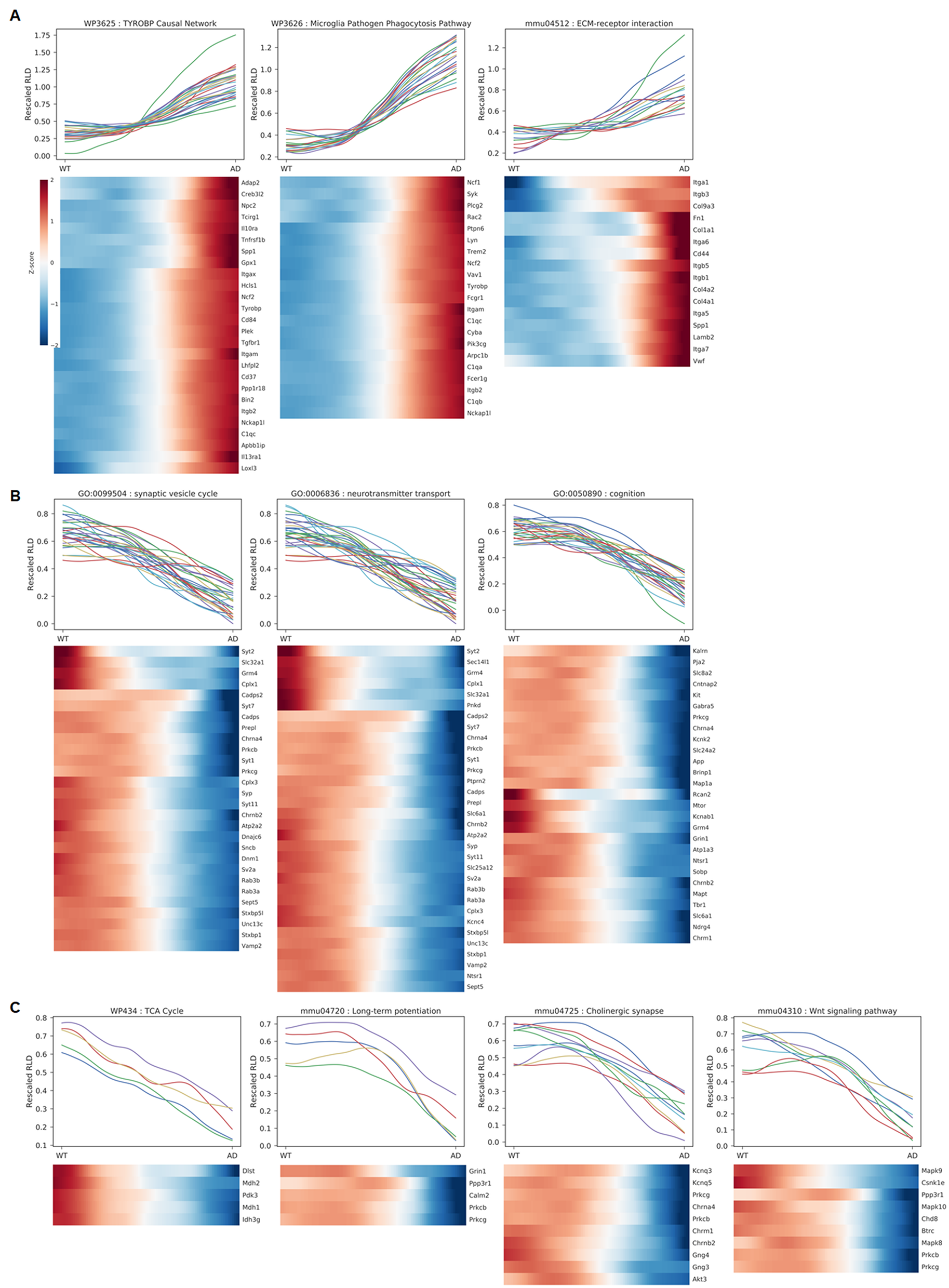

Supplement: S6 Fig — The microglial pathogen phagocytosis pathway shows that most genes follow a gradual increase in P2. The TCA cycle, which is significant in P5, and long-term potentiation, which is significant in P6, have a small number of genes showing homogeneous patterns. Other pathways related to the neurotransmitter transport and vesicle cycle show that the majority of genes follow a gradual decrease (P5) with lower FDR values in P5. However, several genes show early decreases in their transition curves. The pathways related to cognition and cholinergic synapse that many genes follow late decreases (P6) have lower FDR values in P6. (TIF) [file pcbi.1008099.s006.tif]

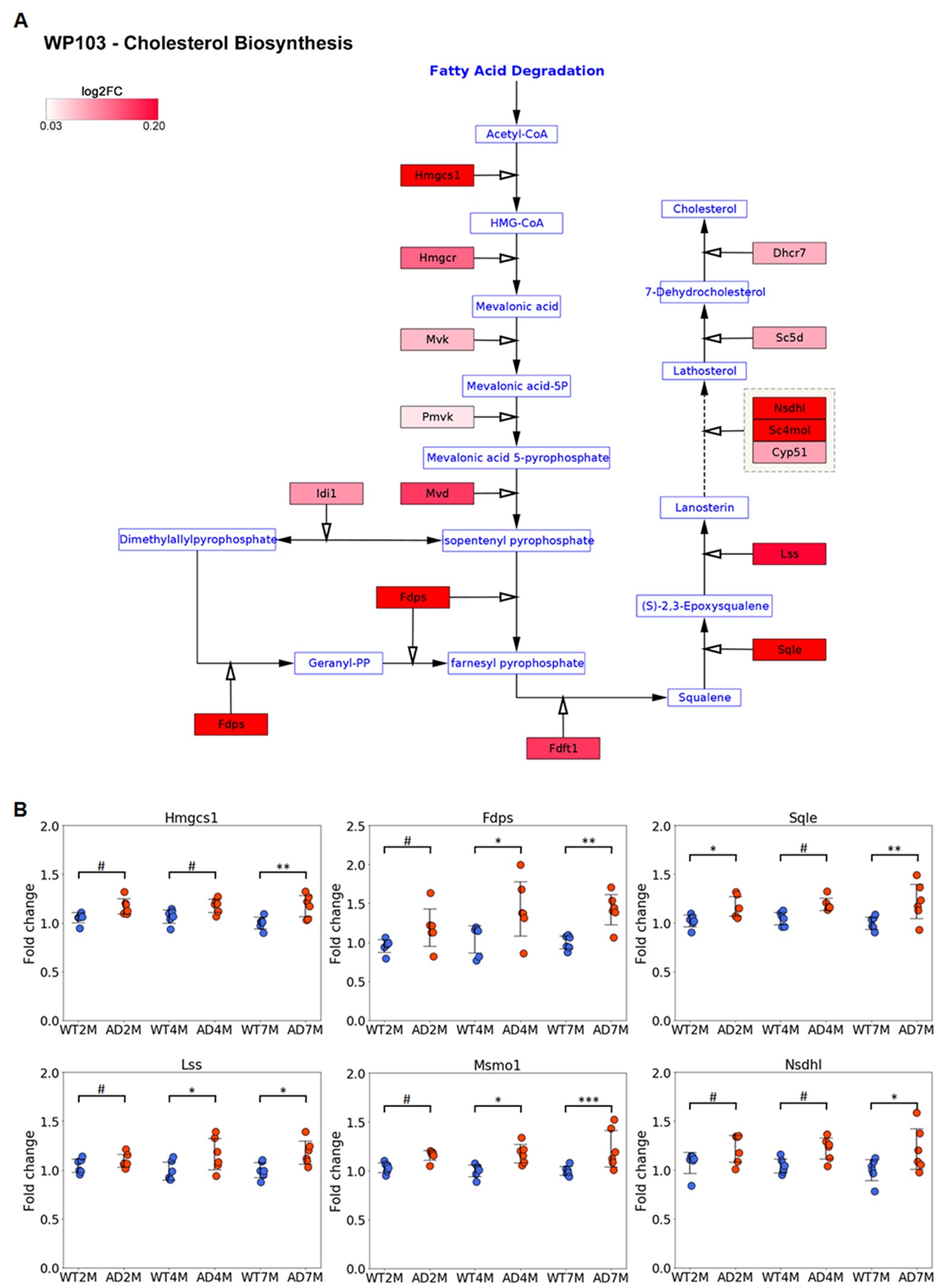

Supplement: S7 Fig — (Top) All 15 genes in cholesterol biosynthesis are colored with log2FC values of AD7M and WT7M. The Sc4mol gene is the same as Msmo1. (Bottom) Plots of the fold changes of six genes that are in the 1,208 DEGs for 7M, in cholesterol biosynthesis. The normalized counts by DESeq2 for gene expression levels are used to evaluate the fold changes against a control value of the mean 7M WT. P-values < 0.1 (#), 0.01(*), 0.001(**) and 0.0001 (***). (TIF) [file pcbi.1008099.s007.tif]

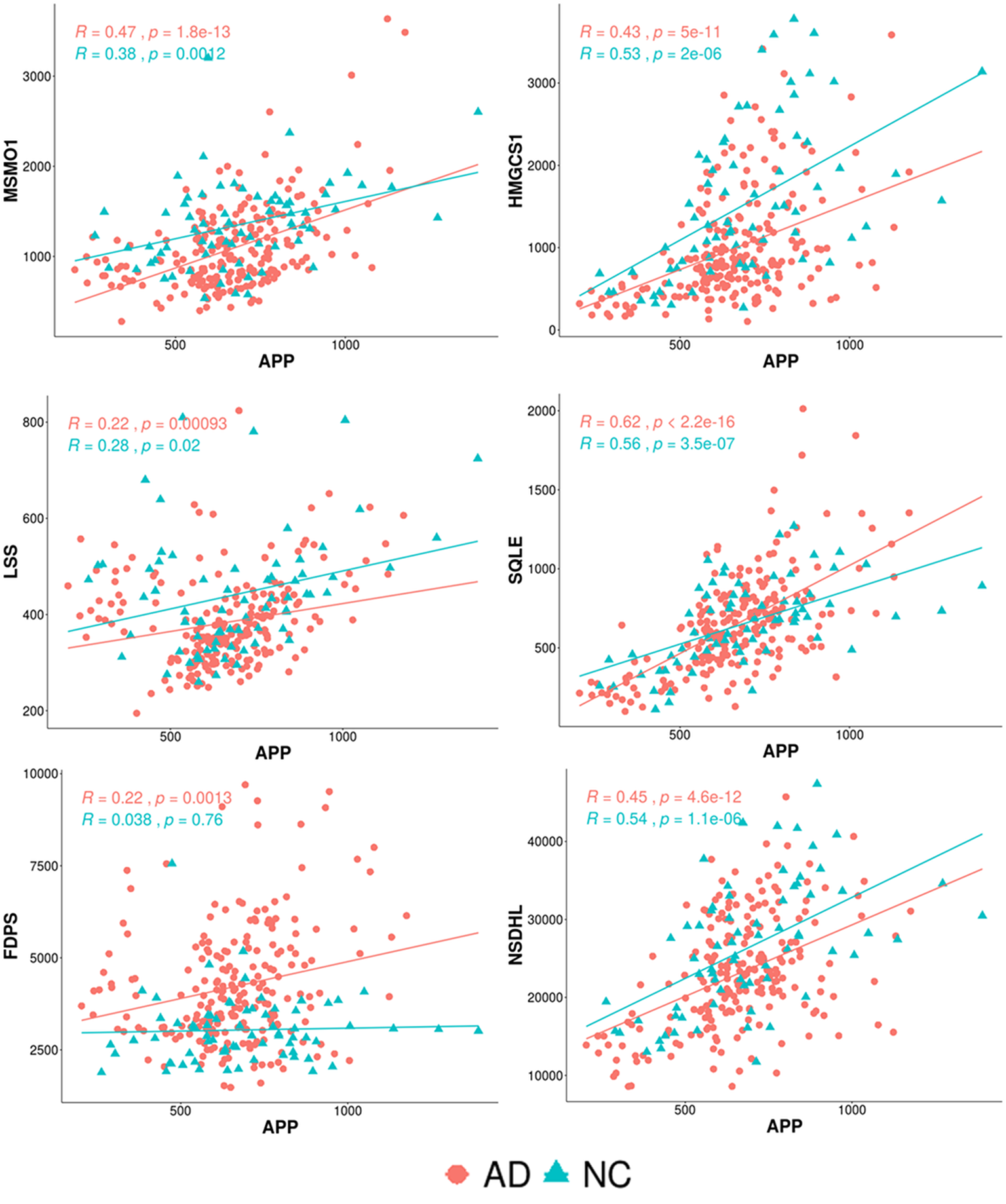

Supplement: S8 Fig — Scatter plots of gene expression levels, which are normalized counts by DESeq2, for six human genes vs human APP. Positive correlations were observed for least four genes with R > 0.4 (MSMO1, HMGCS1, SQLE, and NSDHL). We performed RNA-seq analysis following the pipeline (Trimmomatic-HISAT2-HTSeq2-DESeq2) with GRCh38 annotations. (TIF) [file pcbi.1008099.s008.tif]

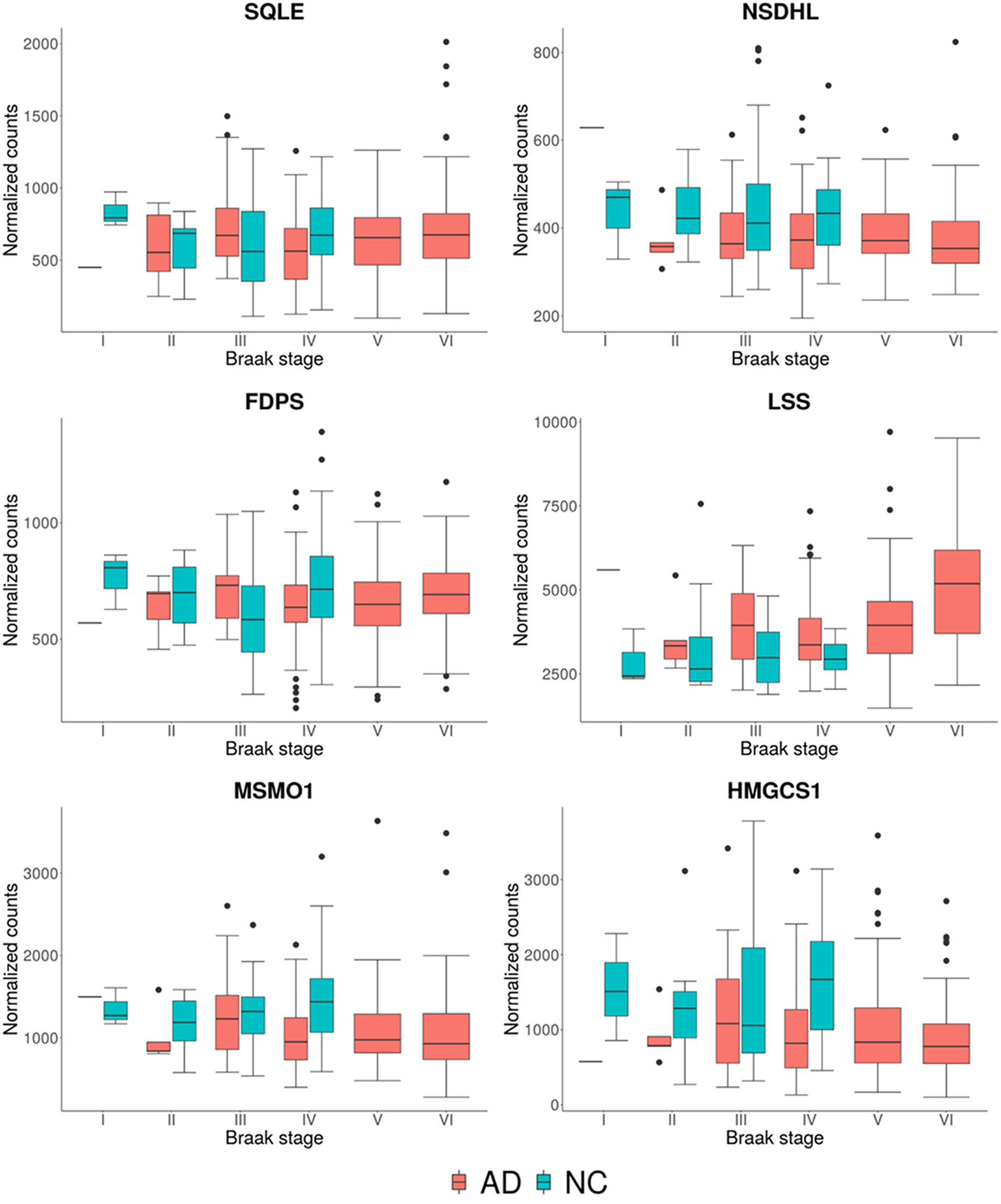

Supplement: S9 Fig — The normalized gene expression levels (TPM) for the six genes in the eight cell-types of mouse cerebral cortex (GSE52564). We performed RNA-seq analysis following the pipeline (Trimmomatic-HISAT2-Stringtie) with GRCm38 annotations. OPC(oligodendrocyte precursor cells), NFO(newly formed oligodendrocytes), MO(myelinating oligodendrocytes). (TIF) [file pcbi.1008099.s009.tif]

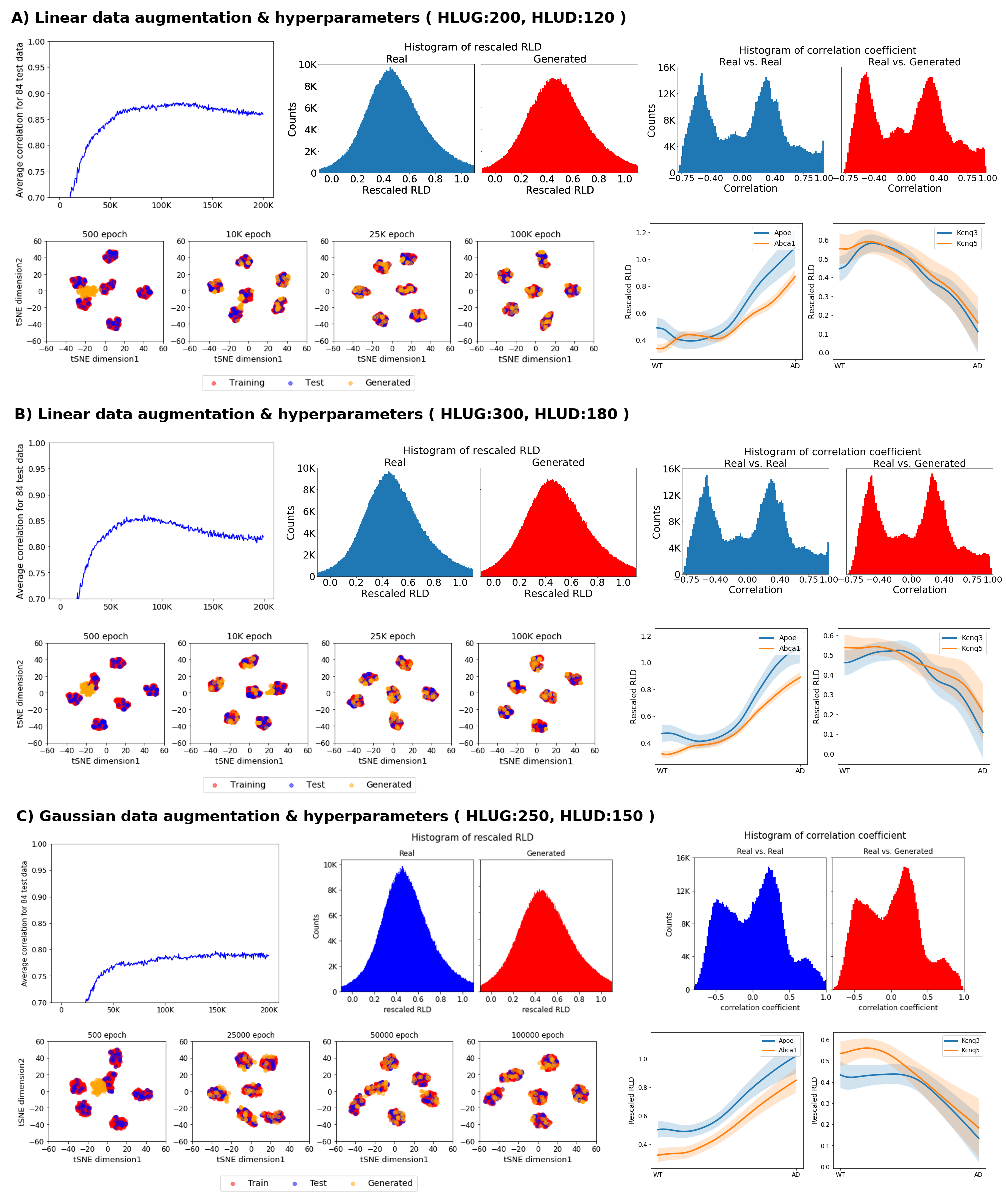

Supplement: S10 Fig — Results of (A) linear data augmentation and hyperparameters (the numbers of hidden layer units of generator (HLUG = 200) and discriminator (HLUD = 120)), (B) linear data augmentation and hyperparameters (HLUG = 300 and HLUD = 18), and (C) gaussian data augmentation and hyperparameters (HLUG = 250. And HLUD = 200). Results are shown by five measures as below. The average pairwise Pearson correlation between 84 generated data and 84 test data corresponding to S1 Fig. The distribution plots of all rescaled RLD values for the 846 augmented real samples (blue) and the 846 generated samples (red) corresponding Fig 2A. Correlation coefficient distributions for all pairs within the 846 real data (blue) and for pairs between the 846 real and 846 fake data (red) at the 100k epoch corresponding Fig 2B. tSNE plots at four epochs with different colored dots representing the 762 training (red) samples, 84 test (blue) samples and 84 generated samples (orange) corresponding Fig 2C. Transition curves of selected four genes from 7M WT to 7M AD corresponding Fig 3A and 3B. (TIF) [file pcbi.1008099.s010.tif]
