# Supplementary material for: A practical application of generative adversarial networks for RNA-seq analysis to predict the molecular progress of Alzheimer's disease
Source: PLoS Comput Biol. 2020 Jul 24;16(7):e1008099. doi: 10.1371/journal.pcbi.1008099 (PMC7406107; doi:10.1371/journal.pcbi.1008099)
Supplement: Attachment — Submitted filename: Response_to_reviewers.docx [file pcbi.1008099.s013.docx]

Decision from PLOS Computational Biology (PCOMPBIOL-D-19-02173) - [EMID:852997389f8e091b]

Dear Dr. Cheon,

Thank you very much for submitting your manuscript "A practical application of generative adversarial networks for RNA-seq analysis to predict the molecular progress of Alzheimer's disease" for consideration at PLOS Computational Biology.

As with all papers reviewed by the journal, your manuscript was reviewed by members of the editorial board and by several independent reviewers. In light of the reviews (below this email), we would like to invite the resubmission of a significantly-revised version that takes into account the reviewers' comments.

Your revision must address all the issues raised by the reviewers and including the points related to reproducibility, robustness and software and data file documentation. Moreover, please be sure to improve the presentation of GANs to a non-expert audience, and to justify their application for gene expression classification.

We cannot make any decision about publication until we have seen the revised manuscript and your response to the reviewers' comments. Your revised manuscript is also likely to be sent to reviewers for further evaluation.

When you are ready to resubmit, please upload the following:

[1] A letter containing a detailed list of your responses to the review comments and a description of the changes you have made in the manuscript. Please note while forming your response, if your article is accepted, you may have the opportunity to make the peer review history publicly available. The record will include editor decision letters (with reviews) and your responses to reviewer comments. If eligible, we will contact you to opt in or out.

[2] Two versions of the revised manuscript: one with either highlights or tracked changes denoting where the text has been changed; the other a clean version (uploaded as the manuscript file).

Important additional instructions are given below your reviewer comments.

Please prepare and submit your revised manuscript within 60 days. If you anticipate any delay, please let us know the expected resubmission date by replying to this email. Please note that revised manuscripts received after the 60-day due date may require evaluation and peer review similar to newly submitted manuscripts.

Thank you again for your submission. We hope that our editorial process has been constructive so far, and we welcome your feedback at any time. Please don't hesitate to contact us if you have any questions or comments.

Sincerely,

Hugues Berry

Associate Editor

PLOS Computational Biology

William Noble

Deputy Editor

PLOS Computational Biology

***********************

Reviewer's Responses to Questions

Comments to the Authors:

Please note here if the review is uploaded as an attachment.

Reviewer #1: This paper presents an application of generative adversarial networks (GANs) to predict the molecular progress of Alzheimer’s disease. It is a topic of interest to researchers in this related field, but the paper needs very significant improvement.

1. In general, there is a lack of explanation of the method of GANs used in this study. The author should provide more detailed information about GANs to make the model clearer for the reader.

Reply: In our previous draft, we tried to suggest a practical application of GANs not to describe detailed methods, which needs too mathematical and technical parameter descriptions. We added a paragraph of general and plain explanations in Materials and Methods section and updated our software at GitHub repository(https://github.com/KBRI-Neuroinformatics/WGAN-for-RNASeq-analysis) with file descriptions (File_Descriptions) and guide readme (README.md) files.

We added a sentence (lines 53-54) as “Brief and general explanations for GANs are given in Materials and Methods.”

We added a paragraph (lines 372-388) in Materials and Methods as

“**Brief explanations of GANs**

GANs are a machine learning algorithm that generate fake data based on given real data. They consist of two models, a generator and a discriminator, which generally use platforms of neural networks. The generator trains its network to generate samples similar to real data. The discriminator tries to distinguish between the generated and the real data as accurately as possible. Both networks perform adversarial learning to optimize their goals based on their loss function. Hence training GANs is a minimax optimization problem. Recently they have been highly paid attention among various generative models due to their continuous performance improvements and powerful applicability via vector arithmetic in latent space. Moreover the generator is thought to capture the semantic features of real data, which enables to generate real-like fake data with a new style that did not exist.

In this study, we try to generate gene expression profiles based on the real profiles for RAN-seq data of 5xFAD mice. The highly resembled fake data are essential to simulate interconversion between two states corresponding to Alzheimer’s disease progression. The detailed network structure, hyperparameters, optimization and analysis procedures of our approaches are given in below sections (Network architecture and hyperparameters, Latent interpolation) and the GitHub repository (https://github.com/KBRI-Neuroinformatics/WGAN-for-RNASeq-analysis).”

2. The results are a little hard to follow for someone who is not an expert in this field or without closely reading the methods.

Reply: Based on many comments of the reviewers, we revised many vague descriptions and make them clearer. We understand it is hard to follow. One of the reasons is that this work is a highly convergence study of AI, bioinformatics and Alzheimer’s disease.

3. Are the results robust? For example, whether different data augmentation method or parameters can lead to large changes in results.

Reply: Yes. Our results are robust to changes in data augmentation methods and variation of hyperparameters. We further perform and present three more cases of GANs trainings and analyses. We added a new figure (S10 Fig) with variations in data augmentation and hyperparameters. We tried to train GANs with different hyperparameter variations of the number of hidden layer units of the generator and the discriminator. We also employed a different data augmentation method, which includes fitting original real data to gaussian distribution and random generation by the gaussian distributions. We checked the average correlation for 84 test data, distributions of rescaled RLD, correlation coefficients of samples, tSNE plots and transition curves for selected four genes. We can see some variations of results showing a little worse performance but roughly all trainings are working very well, which supports our procedure for training GANs is robust.

We added a new figure (S10 Fig) and figure captions as “**S10 Fig. Five measures for robustness checking in variation of GANs trainings.** Results of (A) linear data augmentation and hyperparameters (the numbers of hidden layer units of generator (HLUG=200) and discriminator (HLUD=120)), (B) linear data augmentation and hyperparameters (HLUG=300 and HLUD=18), and (C) gaussian data augmentation and hyperparameters (HLUG=250. And HLUD=200). Results are shown by five measures as below. The average pairwise Pearson correlation between 84 generated data and 84 test data corresponding to S1 Fig. The distribution plots of all rescaled RLD values for the 846 augmented real samples (blue) and the 846 generated samples (red) corresponding Fig.2A. Correlation coefficient distributions for all pairs within the 846 real data (blue) and for pairs between the 846 real and 846 fake data (red) at the 100k epoch corresponding Fig.2B. tSNE plots at four epochs with different colored dots representing the 762 training (red) samples, 84 test (blue) samples and 84 generated samples (orange) corresponding Fig.2C. Transition curves of selected four genes from 7M WT to 7M AD corresponding Fig.3A-B“

We added a section called Robustness of GANs training and analysis procedure in Material and Methods as “**Robustness of GANs training and analysis procedure.** We checked whether our results were robust to changes of data augmentation method and variation of hyperparameters. In this study, we tried a wide variety of hyperparameters to get optimized performance of fake data. As some examples, results with different hyperparameters are shown in S10A-B Fig. It could be seen that GANs training processes were successful and the results were slightly different but overall similar. As mentioned in the above data augmentation section, we devised the data augmentation by linear interpolation, which is simple and easy to apply. However, there may be artifacts, and a different data augmentation method, which uses fitting original real data to gaussian distribution and random generation of augmented data by the distributions, was devised to verify robustness of the procedures. The results are shown in S10C Fig, which shows the training process is working well but the performance is slightly low. Hence we verified robustness of our results to variations of hyperparameters and change of data augmentation method.”

Reviewer #2: Summary:

Authors present in silico study of the molecular mechanism of Alzheimer's disease progression from wild type to disease state using publically available mouse model RNAseq data.

They raise the problem of lacking sufficient experimentally generated data to study various stages of the disease progression and propose to address it by producing synthetic data using generative adversarial networks (GANs) that have become popular deep learning methods in the fields such as image analysis and being adapted for biological studies. In the manuscript authors firstly demonstrate data generation process, then they analyse introduced disease transition curves in the context of disease related pathways. Authors perform pathway enrichment analysis using interpolated data of up- and down-regulated genes and suggest the hypothesis of mutual regulation between amyloid beta production and cholesterol biosynthesis. The manuscript is supported by the analysis code provided via GitHub repository https://github.com/KBRI-Neuroinformatics/GAN-for-bulk-RNAseq.

I am recommending the paper could be accepted after authors have addressed the issues stated in the major and minor comments sections. My major concern is the reproducibility of the results demonstrated in the article. Major and minor comments are provided below.

Major comments:

1. It is not currently mentioned if a power analysis and gene effect sizes were taken to account when identifying a significantly differentially expressed genes.

Reply: We did not take into account a power analysis to determine reliable DEGs based on the sample size, cutoff values of expression levels and fold changes. We used the public data set (GSE104775) and employed all type of genes including protein-coding, non-coding, pseudogenes and etc based on GRCm38.93 gene annotations. When identifying DEGs, our criteria are FDR<0.05 and mean scaled counts > 1000. We did not consider fold change cutoff, since this cutoff greatly reduces the number of DEGs. For our DEGs, power value may be small since the sample size of biological replicates is 6, which is the most critical factor in power analysis. In reality, if new DEGs by power analysis were selected, all analyses in this work should be updated. Anyway, our work is not trying to get reliable DEGs precisely, but rather focusing on inferring expression changes of selected genes between phenotypes. Actually, if we select different gene sets of some pathological pathways ( e.g. immune related pathways) having approximately 1,000 genes, the learning process of GANs is still working but interpretation procedure should be different. Hence the confident DEGs or the sample size are not main topics in this study.

2. It is also not clear whether 1208 DEGs were differentially expressed only between 7MWT vs 7MAD or other comparison was used to identify these genes.

Reply: 1208 DEGs are obtained only between 7MWT vs 7MAD. The description was in the Fig.1 caption but we revised the sentence (line 108) as “we were able to obtain significant DEGs (1,208 genes (7M WT vs 7M AD ), 1,193 genes (4M WT vs 4M AD) and 3 genes (2M WT vs 2M AD) ) filtered by an adjusted p-value < 0.05 (Benjamini-Hochberg procedure) and the DESeq2 scaled counts (the mean scaled counts >100) (S1 Table).”

3. It is not quite clear what causality authors intend to discover on p.4 line 96 and how it is reflected in the results part.

Reply: Our GANs method can provides the sequential orders of gene regulation which inferring pathological cascades. In our paper, we observed the different patterns between cholesterol biosynthesis (early event) and cholesterol metabolism (late event). It suggests that this enhanced cholesterol biosynthesis is not due to cholesterol transportation or esterification but due to direct correlation with enhanced amyloid-beta production. In this way, the sequential orders can distinguish between a cause and results. We added a sentence (line 98-99) as “As an example, we dissect cholesterol biosynthesis as an early event due to enhanced amyloid-beta production.”

4. P.5 line 112; p.19 line 430 Please clarify whether during the augmentation procedure the interpolation was performed between samples belonging to one group or from different groups. Figure 1 caption is a missing explanation of what s1...s2 is.

Reply: The augmentation procedure was performed between samples belonging to one group. We changed the description of data augmentation with notations S1 and S1. We revised the data augmentation section (lines 391-397) in Materials and Methods as “The real data set consists of a total 36 samples from six groups and each group consists of six samples. The data augmentation procedure was initiated based on the pairwise combinations within six samples belonging to a group. 15 pairwise combinations can be considered within six samples. Under a combination between two original samples (S1 and S2) (Fig.1), we created nine augmented samples (S_aug_) by linear interpolation (S_aug_=*x*S1+(1-*x*)S2, where *x*=0.1,0.2,…,0.9 ). Hence a total of 810 augmented samples (6 groups × 15 pairs × 9 linear interpolations) could be produced. Now we can use 846 samples (36 real + 810 augmented samples) for GANs training.”

5. Please clarify the choice of data generated at 100k epoch for the comparison with the original augmented data instead of data generated at 25k epoch when convergence has already been reached.

Reply: We revised two sentences (lines 124-130) as

“We observed that training convergence was reached after 25k epochs (loss values from S1A Fig) and similarity between the generated fake data and the real data has reached a maximum after 75k epochs and has been saturated (S1B Fig).

A comparison of the distribution plots of the normalized gene expression values (rescaled RLD) for the same number of fake samples as the 846 augmented real samples at 100k epoch, where the similarity of the generated data is after the maximum, shows that two distributions are almost identical (Fig 2A), which indicates that our new normalization working very well.“

6. Model performance evaluation metrics such as precision, recall and F1 score are not presented.

Reply: A typical generative model uses an unlabeled learning, and does not directly apply quantitative performance evaluation methods for labeled supervised learning models. Quantitative evaluation by precision, recall and F1 measures for GANs was suggested in 2018 at two preprint drafts (Assessing Generative Models via Precision and Recall (arXiv:2018) and Are GANs Created Equal? A Large-Scale Study (NeurIPS:2018)). However, since the fake data are generated by random variables in our WGAN model, it is necessary to find the similarity measurement to determine which class the generated data belongs to. Hence the precision and recall measurements is again reduced to an approximation problem defining manifolds and evaluating distances from manifolds. In fact, the main difficulty in GANs models is mode collapsing or mode dropping problem, for which the performance evaluation is required to find optimal parameters by optimization process. When mode dropping occurs, precision value may be high but recall value can be low. In this study, we did not use model performance evaluation by precision, recall and F1 score, since we are not intended to focus on model optimization process although we did it by try and error. However, the qualitative performance evaluation is shown in the presented figures. Fig.2D shows the scatterplots of gene expression values between real and resembled fakes which can be corresponding to precision of the model. Fig.2C of tSNE plots and Fig.2E showing expression values of a gene (Apoe) for 36 original real samples indicate that the mode collapsing is not occurring, and our trained model can generate all modes (groups).

7. Please explain why only 846 samples were generated by GANs.

Reply: The data augmentation procedure by linear interpolation produced the 846 samples for GANs training. Hence we generated 846 fake samples only for plotting Fig.2A and 2B at 100k epoch to compare the distributions of rescaled RLD and correlations, which need the same number of samples for comparison. We generated 84 samples for t-SNE plots (Fig.2C) and correlations with test data (S1B Fig.).

We revised a sentence (lines 127-128) as “A comparison of the distribution plots of the normalized gene expression values (rescaled RLD) for the same number of fake samples as the 846 augmented real samples at 100k epoch, ”

8. P.8 line186 - p.9 line193. It is quite challenging to evaluate the claims that authors make about the gene-specific features due to the construction of the sentences. Figure S3 that is referred to as S3A and S3B in fact does not have A and B part, legend and caption.

Reply: We changed S3A and S3B to S3 Fig Top panels and S3 Fig Bottom in lines 165 and 168. We explain the description of the gene-specific features in the reply of the 9^th^ comment.

9. It’s also not clear how collective behavior in the gene association network is connected with the weight parameters of the last layer in the generator.

Reply: We followed the idea of the previous paper by Ghahramani et al suggesting GAN-derived gene association network having features of non-linearly combined co-regulated genes. They could target a new gene of their association network and validated by the ChIP-seq experiment confirming a new type of gene association. We revised the S4 Fig caption (lines 543-551) as “In this way, we observed genes varying in the same pattern over the epochs, and they represent the properties of a sample rather than the properties between the genes. This indicates that the collective behavior of gene expressions were supposed to be learned and optimized to recognize sample-specific features rather than gene-specific features. **(Bottom)** The weight parameters of the last layer in the generator are supposed to contain gene-specific features as suggested by Ghahramani *et al*. in the scRNA-seq study. They suggested this GAN-derived gene association network is corresponding to non-linearly combined co-regulated genes and is distinct from linear and directly correlated regulations of a gene expression profile. Therefore, the correlation heatmap constructed by the weight parameters is predicted to show biological gene associations.”

10. P.9 line 209. It is not clear why only transition curves from 7M WT to 7M AD were selected for further analysis. Also the caption of Figure 3(A-B) is misleading, i.e. it does not provide information that only transition curves from 7M WT to 7M AD were plotted. Please clarify what is meant by “original data points for all months” on Figure S4.

Reply: Since we added a new S2 Fig in revision, S4 Fig becomes S5 Fig. We want to simulate disease progression from 7M WT to 7M AD states, since it is well known that the phenotypes of older 5xFAD mice are clear in their behaviors and brain images with amyloid plagues. The number of DEGs between 7M WT and 7M AD is 1,208 and we applied the GANs to the gene expression profile with only 1,208 DEGs. That is why we focus on transition curves of 1,208 DEGs from 7M WT to 7M AD for further study. Transition curves for 1,208 genes were estimated on three axes (7M WT to 7M AD, 4M WT to 4M AD, and 2M WT to 2M AD). We could not show all transition curves, instead we plotted them of only selected 17 genes for one axis from 7M WT to 7M AD (Fig. 3A-B) and for three axes (S5 Fig). “The original data points for all months” represent the RLD values of the 36 original samples.

We revised sentences (lines 190-193) as “By averaging the procedure over the same states and epochs, we obtained the transition curves for 1,208 genes from WT to AD for 2M, 4M and 7M (see Materials and Methods). For examples, we selected only 17 genes, which are known to be highly related or have similar names, for plotting their transition curves on one axis from 7M WT to 7M AD (Fig 3A–B) and three axes (2M WT to 2M AD, 4M WT to 4M AD, and 7M WT to 7M AD) (S5 Fig). We present the transition curves in RLD scale for 17 genes with the expression values for real 36 samples (three colored dots).”

We revised the figure caption of Fig. 3 as “(A-B) Transition curves of selected 17 genes from 7M WT to 7M AD”

11. For transition curves classification authors use assumption of the existence of 6 predefined disease trajectories. It is not clear if this assumption is based on the previously confirmed domain knowledge or authors propose their hypothesis.

Reply: Based on all transition curves, we proposed the six predefined curves (red colored lines).

We revised the figure caption of Fig.3 as “(C) Each curve belongs to a pattern when r (the correlation coefficient with the predefined red colored curve patterns which we proposed) is higher than 0.95 or when the maximum r is above 0.90;”

12. It’s not clear what is TYROBP causal network is and how it was identified.

Reply: It was suggested as genetic networks in late-onset Alzheimer’s disease in 2013, which is cited as reference 26 (line 237). This pathway can be identified by WikiPathway and our dissection is shown in Fig. 4.

Software:

1. The software is not sufficiently documented to fully reproduce the analysis. I encourage authors to add user guidelines and requirements (e.g. python 3, etc.) to the README file.

Reply: We revised README.md (<https://github.com/KBRI-Neuroinformatics/WGAN-for-RNASeq-analysis/blob/master/README.md>) file in our GitHub repository, where we added required packages and guidelines for training networks and data analysis.

2. Instead of referring to the original dataset GSE104775 from the dedicated repository authors provide their own composed data file GSE104775_RLD_OurDEG_ADWT_bM100_augmentation_rev.npz that is not accompanied by a clarification of what is its content. I suggest authors to add a corresponding description of the data file deposited at github repository.

Reply: Npz format is numpy compressed file having 846 augmented sample profiles of RLD scale. We revised the ‘main.py’ and the jupyter notebook files (WGAN_for_rnaseq_analysis.ipynb), which now starts from the original RLD file (csv format) and includes the augmentation procedure. Now this jupyter file was coded to be simple by calling functions in several python codes. While this jupyter file includes data augmentation, training GANS and other analyses, ‘main.py’ code can be executed by itself till training GANs. We also added a file for describing all code and data files.

3. The file GAN_for_bulkRNAseq.ipynb does not provide differential expression analysis that is the first step of the analysis described in the manuscript. I encourage authors to add missing analyses scripts to fully reproduce the results starting from the downloading of the raw data, preprocessing and differential expression analysis.

Reply: We added script and data files for RNA-seq plipeline (DESeq2_htseq and DESeq2_htseq/RNA-seq_scripts) including a DESeq2 R program, a Hisat2 alignment script, a HTSeq script, a Trimmomatic script files and other minor files, which we used in this study.

4. Analyses of GSE90693 and GSE125583 data sets used for the validation of biological findings are missing from the project repository.

Reply : We uploaded the DEG result files for two additional datasets in WGAN-for-RNASeq-analysis/GSE90693_GSE125583. Since we used the same RNA-seq packages (Trimmomatic, Hisat2, HTseq, DESeq2) as the case of GSE104775, we did not upload the scripts files.

Minor comments:

1. In order to increase the reproducibility of the results I suggest the authors to share software on GitHub with specified release version and accompanied by a code DOI. It can be obtained at DOI providing repository such as Zenodo or similar. This would provide permanent access to a usable instance of the published code. Code with an assigned DOI may be formally cited in future publications.

Reply: We obtained a code DOI (10.5281/zenodo.3784200) of zenodo, which is included in README.md

2. Finally, I noticed the study source code at https://github.com/KBRI-Neuroinformatics/GAN-for-bulk-RNAseq was not available under an open source license. An open source license is essential to allow other researchers to modify and reuse the code. I recommend the authors release the software under a permissive open source license, such as the MIT License. See https://help.github.com/en/github/creating-cloning-and-archiving-repositories/licensing-a-repository.

Reply: We created the new repository (http://github.com/KBRI-Neuroinformatics/WGAN-for-RNASeq-analysis) and added the MIT license following the reviewer’s suggestion.

3. I recommend authors to carefully read their text and pay attention to punctuation.

I found few typos cases where the references were placed after the end of the sentence, e.g. p.3 line 44, 53, 54, 56; p.4 line 78, 90,105, etc. A dot is missing the end of the sentence on p. 9 line 193. Typo on p.13 line 27.

Reply: We corrected all reference places and dot missings.

4. I haven't quite understood the meaning of the sentence on p. 1 line 20-24 in the context of information given in the abstract.

Reply: We changed two sentences in the abstract (lines 20-24) from “The coexpression features are trained well in the network to generate specific sample profiles based on weight parameters with biological gene associations. Virtual disease progress was introduced by latent space interpolation, which has been a key method for various image conversions in GANs, to yield the transition curves of various genes with pathological changes from normal to AD state.” to “We examined how the generator is featured to have specific-sample generation and biological gene association. Based on the above observations, we suggested virtual disease progress by latent space interpolation to yield the transition curves of various genes with pathological changes from normal to AD state.”

Reviewer #3: The authors used generative adversarial networks (GANs) to capture the gene expression transition patterns from wild-type samples (WT) to Alzheimer’s disease (AD) samples. To train the deep neural net, the author did data augmentation and selected differentially expressed genes as the features. Evaluation methods, including Pearson correlations between real and fake samples, distribution of the fake samples compared to real samples, and a t-SNE clustering after training convergence, were used to demonstrate the validity of the augmented samples. The author used the latent space interpolation of the GANs and showed 6 patterns of AD progression, which provide new insights in dissecting biological (AD) pathways. In general, the manuscript is well organized and the study is rigorous, but we have a few concerns:

1. It would be more convincing by showing additional t-SNE clustering results for the original 36 samples and the augmented samples and mind: If either of the t-SNE results shows similar clusters to Figure 2C. If the answer to question (a) is a yes, for every cluster, how many of the genes overlap with those when use all samples including real, augmented and GAN generated samples?

Reply: We performed tSNE plots with the only original 36 samples (S2A Fig), the only augmented 810 samples (S2B Fig), and combined data of original(36)+augmented(810)+resembled fakes(36) (S2C-D Fig). The tSNE plot made by the only original 36 samples do not show any clear clustering. But we can observe clear groups on the tSNE plot with the only augmented samples. Hence the procedure of pairwise data augmentation is expected to enhance the ability of clustered visualization between different groups. The tSNE plot of the combined data show the excellent clustering of the same groups regardless of data sources such as the original, the augmented or the fake samples. Furthermore, the resembled fakes are well overlapped to their corresponding the original 36 samples. Since we only selected 1,208 DEGs (7M WT vs 7M AD) for training GANs, we did not present tSNE plots with marker genes showing clustering features as the way Ghahramani *et al*. did. Gene overlaps can be indirectly shown in the scatterplots for the 1,208 genes between 7M AD samples vs the corresponding resembled fakes (Fig. 2D).

We added a sentence (lines 153-156) as “The additional tSNE plots for the original(36), the augmented(810) and the resembled fakes(36) corresponding to the original samples show that visualization of clustering is enhanced by pairwise data augmentation within a group and the resembled fakes are well overlapped to the original real samples (S2 Fig).”

We added a new figure (S2 Fig) of additional tSNE plots and figure captions as “**Clustering patterns of tSNE plots**. (A) The tSNE plot made by the only original 36 samples shows the scattered sample points without clustering. (B) The tSNE plot made by the only augmented 810 samples shows the clear six groups representing different phenotypes. (C) The tSNE plots of the combined data (the original(36), the augmented(810) and the resembled fakes(36)). The original and the augmented 846 samples are visualized showing excellent clustering of the same groups. (D) The tSNE plot of the combined data including the resembled fakes (black stars) shows well overlaps between the original samples and resembled fakes.”

2. By performing the analysis mentioned above, I would be either convinced or skeptical about the necessity of using GANs to find the patterns, though GANs can make the transition plots.

Reply: The above tSNE plots show the original, the augmented samples and the resembled fakes are well clustered based on their contextual groups. Hence the latent space interpolation between different groups with the resembled fakes seems very reasonable.

3. In the paper, the author put forward the current problem of limited availability of biological samples. But it’s not their deep learning model that solved this problem. What indeed deals with this problem is the process of data augmentation in their method. There might be a misleading claim in the paper that their deep learning model solved the limited sample size problem.

Reply: We suggested a simple data augmentation procedure to overcome untrainability of neural networks with a small number of samples. As we mentioned in line 80-87, the semantic features extracting from training GANs based on the small number of real original samples may show low reliability. Therefore, we focused on generating fake samples that are as similar to the original samples as possible. That is one of the reasons we used a limited number of genes (1,208 DEGs) for training GANs. Hence our work is still limited not covering all gene profiles. Instead of manipulating all phenotype features, we presented an application point using limited samples. In order to avoiding the misleading claim that the problem was solved, the following sentences were modified.

We deleted a phrase “when the sample size is limited” in the abstract (line 30) and changed the sentence from “Here, we suggest that GANs are a useful tool to study disease progression when the sample size is limited, leading to the identification of early pathological signatures.” to “Here, we suggest that GANs are a useful tool to study disease progression, leading to the identification of early pathological signatures.”

We revised a sentence (lines 69-71) by adding a word “transient” as “Nonetheless, it is worthwhile to develop a transient methodology to circumvent this obstacle under the expectation that we will have access to high-quality data in the future.”

We added a sentence (lines 356-358) as “In order to utilize more genes, definitely, more high-quality data with systematic data augmentation in addition to GANs methodological improvements are necessary”

4. The author built a GANs model to simulate gene expression data of 846 real expression data. After showing their fake data simulates the real data pretty well, they tried to interpret the generative network. From their learned model, by using latent space interpolation method in GANs, they were able to classify 6 patterns of gene expression change from WT to AD state. Then they did downstream analysis like pathway analysis to learn the pathological progress of Alzheimer’s disease. But why GANs? Traidtional1. bioinformatics tools could also classify gene expression change patterns since they have time series data for both WT and AD mice. Here the author failed or didn’t show that GANs is better than bioinformatics method, or using GANs is a necessity.

Reply: Given the time series RNA-seq data such as this GSE104775 data with 2M, 4M, 7M ages, the traditional analysis tries to get DEGs at each age with respect to aged controls. And a clustering method such as WGCNA can be applied to produce time series patterns of modules. The above two methods were also used for the GSE104775 data in the original article. However, the number of DEGs (2M WT vs 2M AD) is too small for enrichment analysis and the modules of WGCNA do not show any significant early biological events or time series patterns. In this work, we do not follow DEGs or WGCNA analyses in aging directions. Instead we try to inspect virtual disease progression from WT to AD by the latent interpolation simulations, which is a new approach only the GANs technique can produce. This is a way to track which part happens first and which part happens later in the transition between the two states.

We revised the paragraph (lines 173-186) as

“Here we can apply the latent interpolation in two directions, which are transitions from 2M to 7M in the direction of aging or from WT to AD in the direction of disease progression. In this study, we focus on the latter transition, which is not accessible from traditional bioinformatics analyses. In the AD mouse model (5xFAD), specific transgened human genes (PSEN1, APP) induce a disease state through increased Aβ production. In other words, these mice tend to develop the disease following specific pathways such as the familial AD pathologies. Here, we need to approach the topic from a new perspective. If we have the final AD gene expression state —e.g., activated immune cells, neurite deficiencies, etc.—, the latent vector arithmetic of the GANs would simulate a virtual disease progression from the WT to the AD state by following the methods of image interpolation [11]. That is why we focus on the transition from WT to AD. Although the AD state is caused by the Aβ overproducing mouse model, the coexpression network of the trained GANs might search for semantic changes that capture the disease progression. Our hope is that this GAN application will describe a virtual disease progression and provide novel perspectives regarding pathological cascades that are hard to find using the typical bioinformatics approach of controlling the aging process [16].”

Have all data underlying the figures and results presented in the manuscript been provided?

Large-scale datasets should be made available via a public repository as described in the PLOS Computational Biology data availability policy, and numerical data that underlies graphs or summary statistics should be provided in spreadsheet form as supporting information.

Reviewer #1: Yes

Reviewer #2: Yes

Reviewer #3: Yes

PLOS authors have the option to publish the peer review history of their article (what does this mean?). If published, this will include your full peer review and any attached files.

If you choose “no”, your identity will remain anonymous but your review may still be made public.

Do you want your identity to be public for this peer review? For information about this choice, including consent withdrawal, please see our Privacy Policy.

Reviewer #1: No

Reviewer #2: No

Reviewer #3: No

Figure Files:

While revising your submission, please upload your figure files to the Preflight Analysis and Conversion Engine (PACE) digital diagnostic tool, https://pacev2.apexcovantage.com. PACE helps ensure that figures meet PLOS requirements. To use PACE, you must first register as a user. Then, login and navigate to the UPLOAD tab, where you will find detailed instructions on how to use the tool. If you encounter any issues or have any questions when using PACE, please email us at figures@plos.org.

Data Requirements:

Please note that, as a condition of publication, PLOS' data policy requires that you make available all data used to draw the conclusions outlined in your manuscript. Data must be deposited in an appropriate repository, included within the body of the manuscript, or uploaded as supporting information. This includes all numerical values that were used to generate graphs, histograms etc.. For an example in PLOS Biology see here: http://www.plosbiology.org/article/info%3Adoi%2F10.1371%2Fjournal.pbio.1001908#s5.

Reproducibility:

To enhance the reproducibility of your results, PLOS recommends that you deposit laboratory protocols in protocols.io, where a protocol can be assigned its own identifier (DOI) such that it can be cited independently in the future. For instructions, please see http://journals.plos.org/compbiol/s/submission-guidelines#loc-materials-and-methods

In compliance with data protection regulations, you may request that we remove your personal registration details at any time. (Remove my information/details). Please contact the publication office if you have any questions.
